# Supplementary figures and images for: Highly Similar Sequences of Mature IgA1 Proteases from Neisseria meningitidis, Neisseria gonorrhoeae and Haemophilus influenzae
Source: Pathogens. 2022 Jun 28;11(7):734. doi: 10.3390/pathogens11070734 (PMC9315783; doi:10.3390/pathogens11070734)

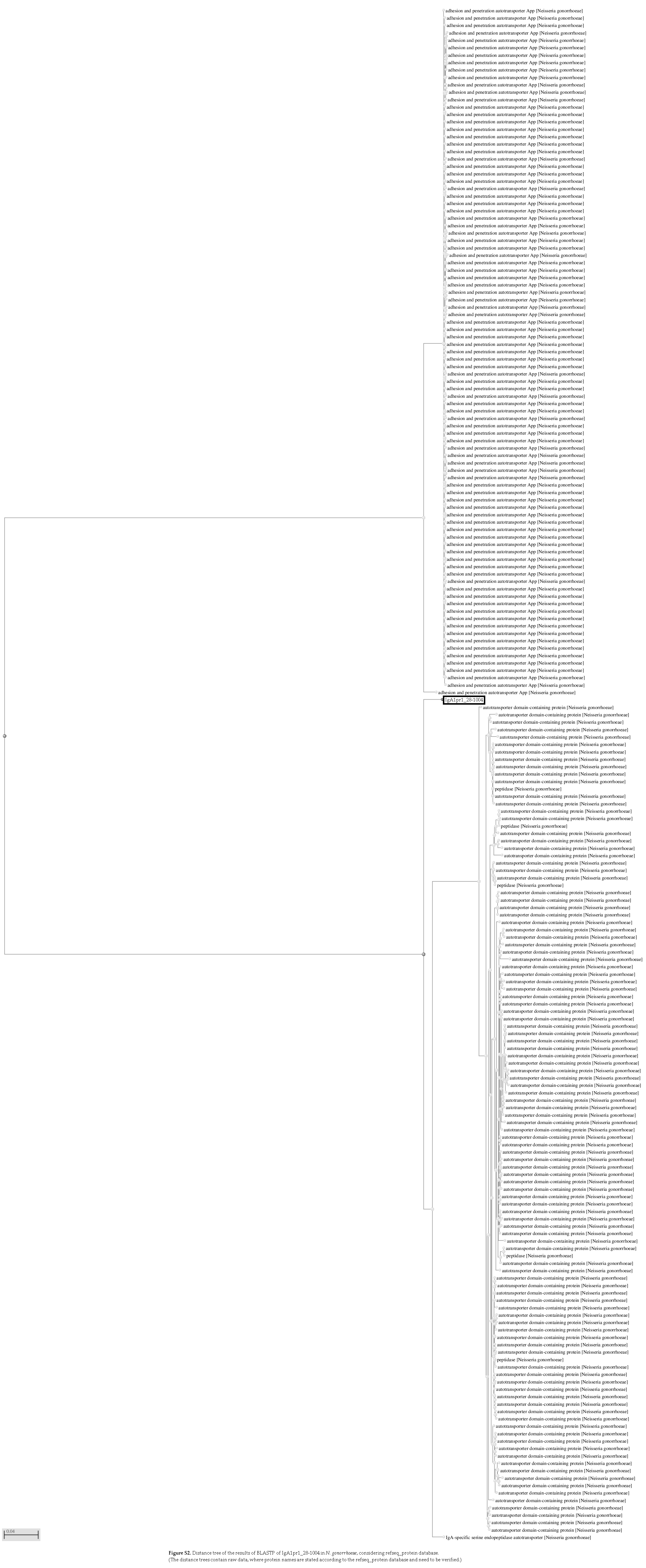

Supplement: Supplementary file 1 [file pathogens-11-00734-s001.zip › Figure S2.png]

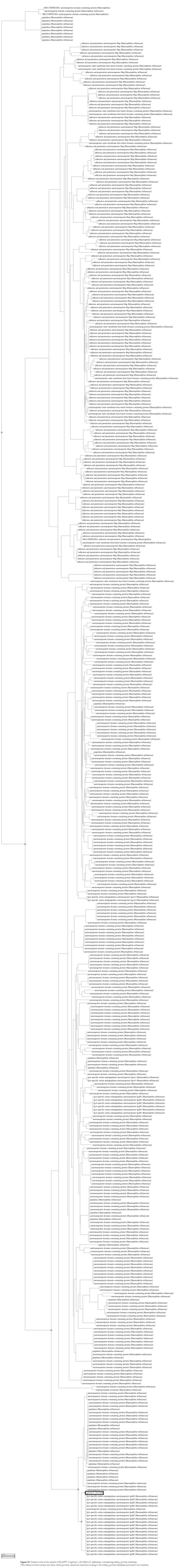

Supplement: Supplementary file 1 [file pathogens-11-00734-s001.zip › Figure S3.png]
